# Association Between Baseline Diastolic Blood Pressure and the Efficacy of Intensive vs Standard Blood Pressure–Lowering Therapy

**DOI:** 10.1001/jamanetworkopen.2021.28980

**Published:** 2021-10-20

**Authors:** Andrew J. Foy, Edward J. Filippone, Eric Schaefer, Matt Nudy, Mohammed Ruzieh, Anne-Marie Dyer, Vernon M. Chinchilli, Gerald V. Naccarelli

**Affiliations:** 1 Department of Medicine, Penn State University Heart and Vascular Institute, Hershey, Pennsylvania; 2 Department of Public Health Sciences, Penn State Milton S. Hershey Medical Center and College of Medicine, Hershey, Pennsylvania; 3 Department of Medicine, Sydney Kimmel Medical Center at Thomas Jefferson University Hospital, Philadelphia, Pennsylvania; 4 Department of Medicine, University of Florida, Gainesville

## Abstract

**Importance:**

Low diastolic blood pressure (DBP) has been found to be associated with increased adverse cardiovascular events; however, it is unknown whether intensifying blood pressure therapy in patients with an already low DBP to achieve a lower systolic blood pressure (SBP) target is safe or effective.

**Objective:**

To evaluate whether there is an association of baseline DBP and intensification of blood pressure–lowering therapy with the outcomes of all-cause death and cardiovascular events.

**Design, Setting, and Participants:**

This cohort study analyzed patients who were randomized to intensive or standard BP control in the Action to Control Cardiovascular Risk in Diabetes–Blood Pressure (ACCORD-BP) trial and Systolic Blood Pressure Intervention Trial (SPRINT). Data were collected from September 1999 to June 2009 (ACCORD-BP) and from October 2010 to August 2015 (SPRINT). Data were analyzed from December 2020 to June 2021.

**Exposures:**

Baseline DBP as a continuous variable.

**Main Outcomes and Measures:**

All-cause death and a composite cardiovascular end point (CVE) that included cardiovascular death, nonfatal myocardial infarction, and nonfatal stroke.

**Results:**

A total of 14 094 patients (mean [SD] age, 66.2 [8.9] years; 8504 [60.4%] men) were included in this analysis. There were significant nonlinear associations between baseline DBP and all-cause death (eg, baseline DBP 50 vs 80 mm Hg: hazard ratio [HR], 1.48; 95% CI, 1.06-2.08; *P* = .02) and the composite CVE (eg, baseline DBP 50 vs 80 mm Hg: HR, 1.45; 95% CI, 1.27-3.04; *P* = .003) observed among all participants. Findings for the interaction between baseline DBP and treatment group assignment for all cause death did not reach statistical significance. For intensive vs standard therapy, the HR of death for a baseline DBP of 50 mm Hg was 1.80 (95% CI, 0.95-3.39; *P* = .07) and that for a baseline DBP of 80 mm Hg was 0.77 (95% CI, 0.59-1.01; *P* = .05). Overall, there was no interaction found between baseline DBP and treatment group assignment for the composite CVE. Over the range of baseline DBP values, significant reductions in the composite CVE for patients assigned to intensive vs standard therapy were found for baseline DBP values of 80 mm Hg (HR, 0.78; 95% CI, 0.62-0.98; *P* = .03) and 90 mm Hg (HR, 0.74; 95% CI, 0.55-0.98; *P* = .04).

**Conclusions and Relevance:**

This pooled cohort study found no evidence of a significant interaction between baseline DBP and treatment intensity for all-cause death or for a composite CVE. These results are hypothesis generating and merit further study.

## Key Points

**Question:**

Is there an interaction between baseline diastolic blood pressure and intensification of blood pressure–lowering therapy for survival and cardiovascular outcomes?

**Findings:**

In this cohort study of 14 094 patients from 2 large randomized clinical trials, baseline DBP of 80 and 90 mm Hg were associated with significant reductions in the risk of a composite cardiovascular end point that included cardiovascular death, nonfatal myocardial infarction, and nonfatal stroke. The same was not true for the outcome of all-cause death.

**Meaning:**

The findings of this study suggest that further work is needed to determine whether blood-pressure lowering therapy should be intensified in patients whose diastolic blood pressure is low.

## Teaser

This cohort study evaluates whether there is an association of baseline diastolic blood pressure (DBP) and intensification of blood pressure–lowering therapy with all-cause death and cardiovascular events.

## Introduction

Since the publication of the landmark Systolic Blood Pressure Intervention Trial (SPRINT),^1^ the focus in treating hypertension has been on systolic blood pressure (SBP). However, this ignores observational data showing a J-shaped curve for diastolic blood pressure (DBP), where adverse cardiovascular events and mortality increase as DBP decreases below critical levels (<60 mm Hg).^2,3,4^ It has been postulated that pharmacological treatment to achieve a lower SBP target in persons whose DBP is already low may worsen patient outcomes. To test this hypothesis, we performed a pooled cohort analysis combining participants from SPRINT^1^ with those of the similarly designed Action to Control Cardiovascular Risk in Diabetes–Blood Pressure (ACCORD-BP) trial^5^ to assess whether there is an association between baseline DBP and treatment intensity on patient outcomes.

## Methods

This cohort study was reported in adherence with the Strengthening the Reporting of Observational Studies in Epidemiology (STROBE) reporting guideline. The study met all criteria for exemption by the institutional review board at Penn State College of Medicine. Informed consent was waived because data were deidentified.

The designs and outcomes of ACCORD-BP^5^ and SPRINT^1^ have been reported previously, and patient-level data from each trial were obtained from the Biologic Specimen and Data Repository Information Coordinating Center of the National Institutes of Health. In brief, ACCORD-BP was a randomized trial of 4733 patients with diabetes with elevated cardiovascular risk assigned to either intensive (ie, SBP <120 mm Hg) or standard (ie, SBP <140 mm Hg) BP control and followed for a median of 4.7 years. SPRINT randomized 9361 patients without diabetes with elevated cardiovascular risk to either intensive or standard BP control, using the same targets as ACCORD-BP, and followed them for a median of 3.3 years.

### Statistical Analysis

We used a Cox proportional hazards model to test the association between baseline DBP as a continuous variable and the outcomes of interest. These outcomes were all-cause mortality and a composite cardiovascular end point (CVE) that included cardiovascular death, nonfatal myocardial infarction (MI), or nonfatal stroke. This composite CVE was chosen because it was the primary composite end point of the ACCORD-BP trial and because each of these individual end points was included in the composite primary end point of the SPRINT trial. The completed analysis included the following variables measured at baseline: DBP, age, sex, history of MI, history of congestive heart failure, history of peripheral vascular disease, history of stroke, estimated glomerular filtration rate at baseline, treatment group (intensive vs nonintensive), stratification factor for study (SPRINT vs ACCORD-BP), and stratification factor for site. To allow for nonlinear associations with outcomes, we used a restricted cubic spline with 4 *df*. The level of significance was set to a 2-sided *P* < .05, and the hazard ratios (HRs) and corresponding 95% CIs were reported from these models. We also showed the association in graphical form using the log hazard scale.

A separate Cox proportional hazards regression model was used to test for an interaction between baseline DBP as a continuous variable and treatment group assignment. The outcomes were all-cause mortality and the aforementioned composite CVE. The completed analysis included all variables used in the model described already but included the additional variable of interaction between treatment group and DBP. To allow for nonlinear associations with outcomes, we used a restricted cubic spline with 4 *df*. The level of significance was set to *P* < .05, and the HRs and corresponding 95% CIs were reported from these models. We also showed the associations in graphical form using the log hazard scale.

For representative purposes, baseline characteristics are presented for all patients stratified by baseline DBP less than 60 mm Hg and greater than or equal to 60 mm Hg. The change in SBP and DBP values from baseline to 12 months is also presented for these DBP subgroups based on treatment group assignment (intensive vs nonintensive).

Analyses were generated using SAS STAT software version 9.4 for Windows (SAS Institute). Data were analyzed from December 2020 to June 2021.

## Results

**Figure 1.**
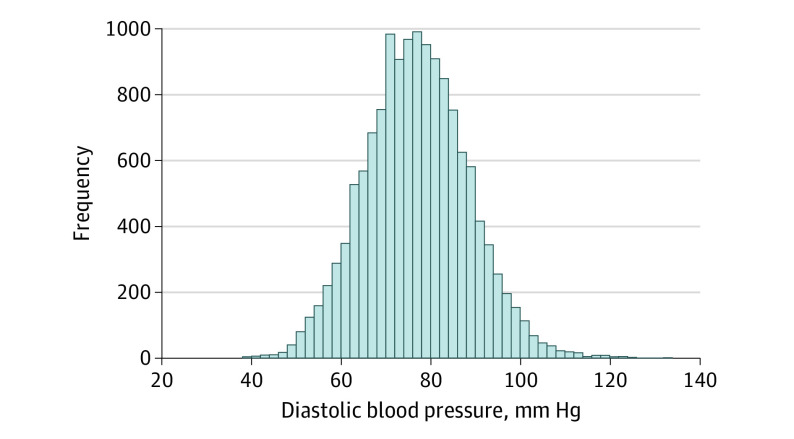
Histogram of Baseline Diastolic Blood Pressure Values Among Participants of the Action to Control Cardiovascular Risk in Diabetes–Blood Pressure and Systolic Blood Pressure Intervention Trial Trials

**Figure 2.**
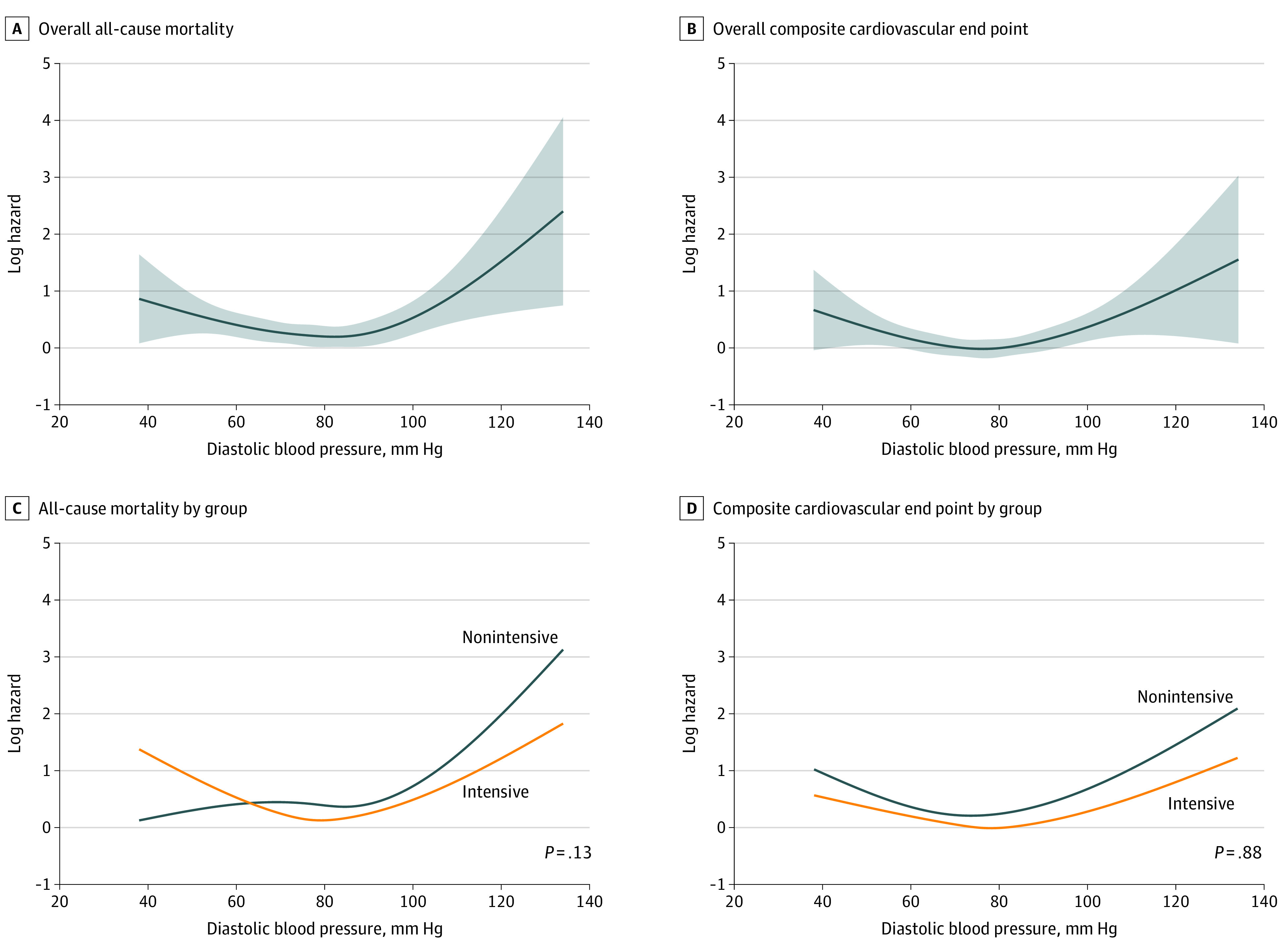
Log Hazard Plots of Baseline Diastolic Blood Pressure and All-Cause Death and the Composite Cardiovascular End Point Log hazard plots of the baseline DBP and all-cause mortality and baseline DBP and the composite cardiovascular end point are shown overall (A and B) and according to treatment group assignment (C and D). Shaded areas in A and B denote 95% CIs.

The data included 14 094 participants (mean [SD] age, 66.2 [8.92] years; 8504 men [60.3%]), with 4733 (33.6%) coming from ACCORD and 9361 (66.4%) coming from SPRINT. The overall number of patients who died was 672 (4.8%), and the number experiencing the combined CVE was 893 (6.3%). More patients died from noncardiovascular causes (392 patients) than cardiovascular causes (280 patients) (Table 1). The median (IQR) baseline DBP was 77 (70-85) mm Hg (Table 1). A distribution plot of baseline DBP values for all participants is provided in Figure 1. In total, 97 patients (0.7%) had at least 1 missing baseline value involving comorbid conditions and were excluded when fitting the models. There were significant associations between baseline DBP (modeled nonlinearly) and all-cause death (eg, baseline DBP 50 vs 80 mm Hg: HR, 1.48; 95% CI, 1.06-2.08; *P* = .02; baseline DBP 110 vs 80 mm Hg: HR, 2.15; 95% CI, 1.30-3.56; *P* = .003) and the composite CVE (eg, baseline DBP 50 vs 80 mm Hg: HR, 1.45; 95% CI, 1.27-3.04; *P* = .003; baseline DPB 110 vs 80 mm Hg: HR, 1.96; 95% CI, 1.27-3.04; *P* = .003). The resulting estimated associations were U-shaped (Figure 2).

**Table 1.** Baseline Characteristics Stratified by Study

Characteristic	Participants, No. (%)		
	ACCORD-BP (n = 4733)	SPRINT (n = 9361)	Total (N = 14 094)
Age at baseline, y			
Mean (SD)	62.7 (6.68)	67.9 (9.40)	66.2 (8.92)
Median (IQR) [range]	62.0 (57.6-67.0) [44.4-79.3]	67.0 (61.0-75.0) [46.0-90.0]	65.0 (59.0-73.0) [44.4-90.0]
Sex			
Male	2475 (52.3)	6029 (64.4)	8504 (60.3)
Female	2258 (47.7)	3332 (35.6)	5590 (39.7)
Diastolic blood pressure, mm Hg			
Participants, No.	4731	9346	14 077
Mean (SD)	76.0 (10.39)	78.1 (11.95)	77.4 (11.49)
Median (IQR) [range]	76.0 (69.0-83.0) [38.0-118.0]	78.0 (70.0-86.0) [40.0-134.0]	77.0 (70.0-85.0) [38.0-134.0]
Congestive heart failure	203 (4.3)	326 (3.5)	529 (3.8)
Peripheral vascular disease	160 (3.4)	503 (5.4)	663 (4.7)
Stroke	307 (6.5)	48 (0.5)	355 (2.5)
Estimated glomerular filtration rate, mL/min/1.73 m^2^			
Participants, No.	4714	9307	14 021
Mean (SD)	91.6 (28.75)	71.8 (20.60)	78.4 (25.45)
Median (IQR) [range]	89.7 (75.5-105.8) [15.3-852.7]	71.4 (58.1-84.7) [14.7-186.2]	77.0 (62.6-91.3) [14.7-852.7]

Abbreviations: ACCORD-BP, Action to Control Cardiovascular Risk in Diabetes–Blood Pressure; SPRINT, Systolic Blood Pressure Intervention Trial.

When analyzed as a continuous variable, findings for the association between baseline DBP and treatment group assignment for the outcome of all-cause death did not reach statistical significance (Figure 2). No interaction was seen between baseline DBP and treatment group for the composite CVE (Figure 2). HRs and corresponding *P* values from the Cox model are provided in Table 2 for intensive vs standard groups.

**Table 2.** Estimated HRs From Cox Models for All-Cause Mortality and the Composite Cardiovascular End Point When Baseline DBP Is Treated as a Continuous Variable

DBP by treatment interaction, intensive vs nonintensive, mm Hg	All-cause mortality		Composite cardiovascular end point	
	HR (95% CI)	*P* value	HR (95% CI)	*P* value
Overall	Not applicable	.13	Not applicable	.88
50	1.80 (0.95-3.39)	.07	0.77 (0.44-1.33)	.35
60	1.12 (0.84-1.50)	.43	0.85 (0.66-1.10)	.22
70	0.82 (0.65-1.02)	.08	0.85 (0.69-1.03)	.10
80	0.77 (0.59-1.01)	.05	0.78 (0.62-0.98)	.03
90	0.85 (0.59-1.20)	.35	0.74 (0.55-0.98)	.04
100	0.79 (0.48-1.28)	.34	0.67 (0.45-1.01)	.05
110	0.64 (0.25-1.64)	.35	0.60 (0.26-1.37)	.22

Abbreviations: DBP, diastolic blood pressure; HR, hazard ratio.

For intensive vs standard therapy, for a baseline DBP of 50 mm Hg, the HR of death was 1.80 (95% CI, 0.95-3.39; *P* = .07); for 60 mm Hg, the HR was 1.12 (95% CI, 0.84-1.50; *P* = .43); for 70 mm Hg, the HR was 0.82 (95% CI, 0.65-1.02; *P* = .08); for 80 mm Hg, the HR was 0.77 (95% CI, 0.59-1.01; *P* = .05); for 90 mm Hg, the HR was 0.85 (95% CI, 0.59-1.20; *P* = .35), for 100 mg Hg, the HR was 0.79 (95% CI, 0.48-1.28; *P* = .34); for 110 mm Hg, the HR was 0.64 (95% CI, 0.25-1.64; *P* = .35) (Table 2). For intensive vs standard therapy, significant reductions in the composite CVE were found for DBP values of 80 mm Hg (HR, 0.78; 95% CI, 0.62-0.98; *P* = .03) and 90 mm Hg (HR, 0.74; 95% CI, 0.55-0.98; *P* = .04) (Table 2).

There were 800 participants with baseline DBP less than 60 mm Hg and 13 277 with baseline DBP greater than or equal to 60 mm Hg. Individuals with baseline DBP less than 60 mm Hg were older (mean [SD] age, 73.6 [8.7] vs 65.7 [8.7] years) and had 3-fold higher risk of dying (74 patients [9.3%] vs 598 patients [4.5%]) than those with baseline DBP greater than or equal to 60 mm Hg (Table 3). Baseline SBP and DBP values were similar for those assigned to intensive vs standard control at baseline in both subgroups; however, by 12 months there were significant differences in achieved levels (Table 3). For individuals with baseline DBP less than 60 mm Hg, the mean (SD) SBP and DBP values at 12 months for the intensive and standard therapy groups were 122.4 (14.9) and 56.3 (9.0) mm Hg and 134.2 (14.9) and 62.4 (9.6) mm Hg, respectively. For those with baseline DBP greater than or equal to 60 mm Hg, the mean (SD) SBP and DBP values at 12 months for the intensive and standard therapy groups were 121.0 (13.8) and 68.8 (9.6) mm Hg and 135.6 (13.7) and 76.0 (10.4) mm Hg, respectively.

**Table 3.** Baseline Characteristics of Individuals With Baseline DBP Less Than and Greater Than or Equal to 60 mm Hg Along With Blood Pressure Control at Baseline and 12 Months

Variable	Participants, No. (%)	
	DBP <60 mm Hg (n = 800)	DBP ≥60 mm Hg (n = 13 277)
Age, y		
Mean (SD)	73.6 (8.7)	65.7 (8.7)
<50	0	111 (0.8)
50-59	68 (8.5)	3629 (27.3)
60-69	183 (22.9)	5379 (40.5)
70-79	343 (42.9)	3210 (24.2)
≥80	206 (25.8)	948 (7.1)
Sex		
Male	472 (59.0)	8026 (60.5)
Female	328 (41.0)	5251 (39.5)
Myocardial infarction	115 (14.4)	1295 (9.8)
Congestive heart failure	46 (5.8)	483 (3.6)
Peripheral vascular disease	88 (11.0)	575 (4.3)
Stroke	29 (3.6)	326 (2.5)
Estimated glomerular filtration rate, mean (SD), mL/min/1.73 m^2^	68.4 (23.7)	79.0 (25.4)
Diabetes		
Uncomplicated	94 (11.8)	2118 (16.0)
End-organ damage	167 (20.9)	2352 (17.7)
BP control, mean (SD), mm Hg		
Baseline		
SBP		
Intensive therapy	127.0 (15.4)	140.2 (15.6)
Standard therapy	128.1 (15.7)	140.3 (15.1)
DBP		
Intensive therapy	54.9 (3.8)	78.8 (10.4)
Standard therapy	55.2 (3.7)	78.7 (10.3)
12 mo		
SBP		
Intensive therapy	122.4 (14.9)	121.0 (13.8)
Standard therapy	134.2 (14.9)	135.6 (13.7)
DBP		
Intensive therapy	56.3 (9.0)	68.8 (9.6)
Standard therapy	62.4 (9.6)	76.0 (10.4)

Abbreviations: BP, blood pressure; DBP, diastolic blood pressure; SBP, systolic blood pressure.

## Discussion

In this pooled cohort analysis of patients from the ACCORD-BP and SPRINT trials, we tested the association between baseline DBP and patient outcomes. We also tested for an association between baseline DBP and treatment group assignment with respect to patient outcomes. Our results confirm prior observations of a nonlinear association between DBP and patient outcomes that appears U-shaped. No statistically significant interactions were observed, however, with respect to DBP and treatment group assignment for all-cause mortality or the composite CVE. This undermines the traditional J-curve hypothesis relating low DBP with reduced coronary perfusion and events.

Although we acknowledge our findings do not establish the existence of an interaction between baseline DBP and treatment group assignment for the outcome of all-cause death, we think it would be imprudent to dismiss possible associations altogether. Treatment effect heterogeneity emerges from a few essential risk dimensions, including (1) the risk of the primary study outcome, (2) competing risk, (3) the risk of treatment-related harm, and (4) direct treatment-effect modification.^6,7,8^ In individuals with lower baseline DBP values, factors unrelated to blood pressure may be associated with death more so than for individuals with higher DBP. Patients whose baseline DBP was less than 60 mm Hg compared with those with higher values were at 3-fold higher risk of dying (9% vs 3%), were significantly older (73.6 vs 65.7 years), and had higher rates of all comorbid conditions (Table 3). It is also not implausible to speculate that such individuals would be at increased risk of treatment-related harm, both measured and unmeasured and that they would not recover the same from adverse events as healthier subjects (adverse events were significantly increased in ACCORD-BP and SPRINT in the intensive therapy groups), and finally, that lowering blood pressure would not yield the same benefits as in those generally healthier subjects. A limitation of this analysis is that it did not look at serious adverse events in relation to baseline DBP, but this was intentional owing to the different definitions and adjudication procedures used for defining these events within the trials.

The notion of treatment effect heterogeneity for common interventions in high-risk patients is not unprecedented in cardiovascular medicine. An example of this within an individual trial can be found in the Sudden Cardiac Death in Heart Failure Trial,^9^ where there was a significant association of heart failure class and implantable cardioverter-defibrillator therapy with mortality. Patients with New York Heart Association class III heart failure derived no benefit from implantable cardioverter-defibrillator therapy but those with New York Heart Association class II heart failure benefited greatly. The 5-year mortality rates in the New York Heart Association classes III and II placebo groups were 46% and 32%, respectively.^9^ This same dynamic can be seen across trials as well. In the Study to Evaluate the Use of Rosuvastatin in Subjects on Regular Hemodialysis trial,^10^ a high-risk population of dialysis patients derived no benefit from statin therapy. The rates of death and nonfatal MI in the control group of this population were 14 per 100 person-years and 2.5 per 100 person-years, respectively.^10^ Contrary to this, in the Justification for the Use of Statins in Prevention: An International Trial Evaluating Rosuvastatin trial,^11^ a much lower risk population of patients who were in generally good health derived a significant benefit from statin therapy. The same rates of death and nonfatal MI in the control group of this population were 1.25 per 100 person-years and 0.33 per 100 person-years, respectively.^11^

In the recently published International Study of Comparative Health Effectiveness with Medical and Invasive Approaches-Chronic Kidney Disease trial,^12,13^ patients with advanced chronic kidney disease derived no benefit from an invasive compared with conservative strategy and were more likely to experience a stroke or a composite of death from any cause or initiation of dialysis. The 3-year rates of death and nonfatal MI in the control group of this population were 27.8% and 15.9%, respectively.^12,13^ In the main trial,^14,15^ which excluded CKD patients with chronic kidney disease, fewer events were experienced in the invasive group, and there was a significant reduction in angina and no evidence of harm. The 3-year rates of death and nonfatal MI in the control group of this population were 4.3% and 8.5%, respectively.^14,15^

Our study is not the first to look for an interaction between intensive therapy and baseline DBP in an intention-to-treat fashion. In a reanalysis of SPRINT-only patients, Beddhu et al^16^ did not find an interaction between baseline DBP and all-cause death, the composite CVE, or chronic kidney disease events when DBP subgroups were analyzed on the basis of quintiles, or when the lowest quintile was compared with the remaining cohort. There are several notable differences between this study^16^ and ours. First, the use of SPRINT-only patients would reduce power to detect any subgroup interactions, if present. Second, their method only considered baseline DBP as a dichotomous variable (based on quintiles) and in doing so, they included many patients with DBPs much greater than 60 mm Hg in their lowest quintile (the mean [SD] DBP of this group was 61 [5] mm Hg).^16^ Our analysis of DBP as a continuous variable suggests the interaction occurs very near the 60 mm Hg mark. Finally, in their analysis they do report a *P* value for the interaction between baseline DBP and treatment intensity on all-cause death of .29.^16^ Although this is far from the traditional bounds of what is considered statistically significant, it may not be trivial. *P* values for testing interactions may be considered hypothesis-generating at lower thresholds than the 95% mark used for hypothesis testing and confirmation.

Li and colleagues^17^ studied the association between DBP and outcomes for patients in the ACCORD-BP and SPRINT trials among patients who achieved a SBP less than 130 mm Hg. Among such patients, those with treated DBP lower than 60 mm Hg had higher rates of events. Their findings are consistent with our study and many others in the past that have looked at baseline and achieved DBP levels and found associations between lower values and outcomes. We did this by looking at baseline DBP as a continuous variable and found significant nonlinear associations with all-cause death and a composite CVE among patients in the ACCORD-BP and SPRINT trials (Figure 2). However, these observations in themselves do not directly address the question of whether starting or intensifying therapy in patients with DBP values less than a certain number to achieve a lower SBP target is effective or not. To the best of our knowledge, only this study and the one by Beddhu et al^16^ address this question directly.

It should not be construed from our data or the other studies mentioned here that patients with low DBP should have deintensification of therapy with the aim of increasing DBP. The question addressed by our study is whether therapy can be safely intensified to lower SBP in such patients.

### Strengths and Limitations

Our study has both strengths and limitations. The main strength is the use of individual patient data from 2 large, randomized clinical trials conducted in the same manner, including both patients with and without diabetes. Limitations include the lack of a significant number of patients with stroke at baseline and the low number of patients whose baseline DBP was less than 60 mm Hg.

## Conclusions

In conclusion, this pooled cohort analysis of patients enrolled in the ACCORD-BP and SPRINT trials supports the existence of a nonlinear association between baseline DBP and patient outcomes as previously reported. More importantly, it suggests that among patients whose baseline DBP was greater than 60 mm Hg, intensifying therapy to achieve an SBP target of 120 mm Hg could reduce death and cardiovascular events. It does not allow us to conclude the same for patients whose baseline DBP is 60 mm Hg or below since intensifying therapy may not reduce the risk of dying and could possibly increase it. This topic merits further investigation.
